# Determinants of male participation in reproductive healthcare services: a cross-sectional study

**DOI:** 10.1186/1742-4755-10-27

**Published:** 2013-05-16

**Authors:** Md Shahjahan, Shirin Jahan Mumu, Afsana Afroz, Hasina Akhter Chowdhury, Russell Kabir, Kapil Ahmed

**Affiliations:** 1 Assistant Professor, Department of Biostatistics, 125/1 Darussalam, Mirpur, Dhaka -1216, Bangladesh; 2 Assistant Professor, Department of Epidemiology, Bangladesh Institute of Health Sciences (BIHS), Darus Salam, Mirpur, Dhaka -1216, Bangladesh; 3 Lecturer, Department of Biostatistics, Bangladesh Institute of Health Sciences (BIHS), Darus Salam, Mirpur, Dhaka -1216, Bangladesh; 4 Doctoral Student, Economics & Statistics Department, Business School, Middlesex University, London, UK; 5 Research Manager, Bangladesh Center for Communication Programs, Dhaka, Bangladesh

**Keywords:** Cross-sectional studies, Male participation, Reproductive health, Bangladesh

## Abstract

**Background:**

The role of male’s participation in reproductive healthcare is now well-recognized. The present study investigated the role of men in some selected reproductive health issues, characterizing their involvement, including factors influencing their participation in reproductive healthcare services.

**Methods:**

This study was conducted in the working areas of urban and rural implemented by NGOs. The sample-size was determined scientifically. The systematic sampling procedure was used for selecting the sample. The study included 615 men aged 25-45 years. Bivariate analysis was performed between male’s involvement as the dependent variable with several independent variables. Logistic regression analysis was applied to assess the effects of risk factors on the participation of men in reproductive health care services.

**Results:**

The mean age of the respondents was little over 34 years while their mean years of schooling was 3.7, and their mean monthly income was about Tk 3,400 (US$ 1 = Tk 70) at the time of the study. Rickshaw-pulling and driving was the main occupation of the respondents from the urban while farming were main occupation in the rural area respectively. About two-thirds of the respondents discussed reproductive health issues with their wives and accompanied them to healthcare facilities. The current contraceptive-use rate was 63% among the men who attended the evening clinics. Results of bivariate analysis showed a significant association with education, occupation, income, access to media, and number of living children. Results of logistic regression analysis showed that secondary to higher education level, number of living children, paid employment status, long marital duration, and access to media were important correlates of males’ involvement in reproductive healthcare services.

**Conclusions:**

The results imply that a greater integration of reproductive healthcare matters with the Millennium Development Goals and increasing perception of men through enrollment in various components of reproductive activities will produce synergistic effects.

## Background

The Program of Action of the ICPD clearly set a new agendum when it emphasized on male’s responsibilities and participation in reproductive healthcare services [1]. Although consensus was reached on involvement of men in reproductive health, and the policy environments generally support that notion in many countries including Bangladesh, healthcare service for reproductive health is still largely female-oriented. The reproductive health programmes have traditionally focused on women and the exclusion of men. However, results of recent studies revealed that men might serve as gatekeepers to women’s access to reproductive health services significantly [2].

The concept of reproductive healthcare is that men, women, and young people have the right to be informed and have access to safe, effective, affordable, and acceptable reproductive healthcare services [3]. Although there is a tendency to overlook the relevance of men in matters relating to reproductive healthcare, they have substantial reproductive health influence. So, reproductive health in its broader sense should be a concern for all, not just that of women.

A large number of articles [4-6] and the growing number of conferences, research projects, and debates on this subject bear testimony to the importance of this issue, both from the programmatic point of view and as a process for bringing about a gender balance in men’s and women’s reproductive rights and responsibilities. This renewed interest in male’s involvement is not unconnected with the HIV/AIDS pandemic that has spurred an intense interest in the promotion of condom-use. Effective family planning is important in spacing childbirth so that both mother and child can gain the maximum quality of life, especially the mothers at high -risk. Birth spacing will also give the mother ample time to recuperate from her previous pregnancy [7].

Men, especially in Africa, are dominant and are the major decision-makers in family affairs, including reproductive healthcare matters [8]. The dominance of male in this respect is reinforced by the cultural institution of patriarchy, religion, and the economic power that men wield. Ezeh reportedted that, in Ghana, spousal influence in respect of reproductive goals, rather than being mutual or reciprocal, is an exclusive right exercised only by the husband [9]. In Ilorin, Nigeria, one of the major reasons for not adopting modern contraceptive method by women is the husband’s resistance [10]. In northern Nigeria, women cannot practice family-planning method without the formal consent of their husbands [11].

Reasons for involving men in reproductive health matters are multifaceted. First of all, men have their own reproductive health concerns and their involvement should not be seen only as a means to achieve women’s better reproductive healthcare. Second, men’s sexual and reproductive well-being and behaviours directly affect their partners. Third, decisions on the matters of reproductive healthcare occur within relationships that affect both men and women [3]. The involvement of men in reproductive healthcare matters should be seen as an important measure for achieving the MDGs that include the reduction of maternal mortality and reducing the prevalence and impact of HIV/AIDS [12].

In the present context of Bangladesh, involving men and bringing positive influences in reproductive healthcare services are the crucial aspects of enhancement of couples’ reproductive healthcare services. Therefore, identification of demographic variables relating to men’s involvement and the contribution of different influencing factors to demographic change would help to formulate future policies for achieving the demographic target through men’s involvement. In this paper, an attempt has been made to assess the relationship between the level of men’s involvement and the demographic variables in order to measure the contribution of different factors for increasing the participation of men in reproductive activities.

## Methods

### Operational definition

#### Male involvement

Among the spousal communicating men, those who are visiting clinics with their wives, and of them, those attend delivery care are considered that they participate or are involved in reproductive healthcare services. Towards ensuring an effective participation of males in reproductive healthcare services, spousal communication of men, accompanying wives during visits to clinics, and their delivery care are the essential preconditions of male’s involvement.

#### Evening clinic

Evening clinics are those which are run by Service providers and which offer counseling on reproductive health issues for males in the evening (from 5 pm to 9 pm).

### Methods and procedures

This cross-sectional study was carried out among males who visited some selected NGOs working in both urban slums and rural areas of Bangladesh. Married males who attended an evening clinic constituted the sampling frame. In total, 615 men were randomly selected for the study.

The sample-size^*^ was determined using the statistical cluster-sampling technique. The cluster was NGO evening clinics. Six study sites were randomly selected from NGOs working in urban slums and rural areas located in Agargoan (Dhaka), Narayanganj, Narsingdi, Tangail, Narail, and Gaibandha. From each of these six sites, at least 100 men were interviewed employing a systematic sampling technique. Trained interviewers and field supervisors were recruited to collect data from the health centers. A pre-tested structured questionnaire was used for collecting information on socio-demographic characteristics, cultural factors, and on the use of family-planning methods.

### Analysis of data

Data were analyzed using the SPSS software for Windows (version 17). The associations between the variables were measured using the appropriate statistical techniques such as χ^2^ and logistic regression. Bivariate analysis was performed between male’s involvement as the dependent variable and each independent variable. Linear logistic regression analysis was done to determine the factors affecting men’s participation in reproductive healthcare services.

^*^The sample size was determined using the formula:

$$n={\mathrm{NZ}}^{2}p\left(1-p\right)/{\mathrm{Nd}}^{2}+Z^{2}p\left(1-p\right)$$

## Results

The distribution of the socioeconomic and demographic characteristics of the respondents is shown in Table 1. The mean age of the respondents was 34 [standard deviation (SD) ± 7.6)] years. Forty-four percent of the respondents had no education. The mean years of schooling of the respondents were 3.7 (SD ± 4.1). Rickshaw-pulling and driving were the primary occupations of the men living in the urban slums, followed by business, monthly salaried job, and day laborer. The mean income was Tk 3,438 (US$ 1 = Tk 70), and the mean land holding was 37.5 decimals. In the case of access to media, around 34% of the men had no access to any media, 35% had access to one, 21% to any two, and 10% had access to all three media, such as newspapers, radio, and television (TV).

**Table 1:** Socio-demographic characteristics of respondents (n = 615)

**Variable**	**Number**	**Percentage**
*Age (years)*		
*≤30*	*231*	*37.6*
31-40	268	43.6
41+	116	18.8
(mean; 34.1 SD = ±7.6)		
*Education*		
No education	272	44.2
Up to class Five	132	21.5
Up to HSC pass	201	32.7
Graduate and above	10	01.6
(mean; 3.7 years, SD = ±4.1)		
*Occupation*		
Farming	263	42.7
Service	91	14.8
Business	198	32.2
Driving	63	10.3
*Access to media*		
*Read newspapers*		
Yes	118	19.2
No	497	80.8
*Listening radio*		
Yes	315	51.2
No	300	48.8
*Watching television*		
Yes	227	36.9
No	388	63.1
*Marital duration (years)*		
<5	177	28.8
5-10	183	29.8
11+	255	41.4
*Wife work outside*		
Yes	56	9.1
No	559	90.9
*No. of living children (n = 531)*		
1	158	30.8
2	154	30.0
3	108	21.0
4+	93	18.2
(mean = 2; SD ± 1.6)		

Results are present as n (%) and mean (±SD). HSC: Higher Secondary Certificate.

The distribution of respondents’ inter-spousal communication is shown in Table 2. More than half (58%) of the husbands accompanied their wives during visits to clinics. Two-thirds of the husbands discussed about reproductive healthcare issues with their wives. Most (95%) couples were approving any family-planning methods. The proportion of couple currently using any contraceptive method was 63.1%.

**Table 2:** Distribution of respondents by their inter-spousal communication

**Variable**	**Number**	**Percentage**
*Visits to clinics with wife*		
Yes	354	58.4
No	252	41.6
*Discussing about reproductive health issues*		
Yes	416	66.0
No	197	32.0
Others	12	2.0
*Currently using family planning methods*		
Yes	388	63.1
No		
*Approval of family planning*	227	39.9
Yes	585	95.1
No	30	4.9

Results are present as n (%).

Table 3 presents the results of bivariate analysis between the male’s involvement and the demographic variables. A significant association was found among education (p < 0.001), occupation (p < 0.001), income (p < 0.001), access to media (p < 0.001), and number of living children (p < 0.003).

**Table 3:** Bivariate analysis between male’s involvement and demographic variables

**Background characteristics**	**Male’s involvement in reproductive health**		**p value**
	**Yes (n = 152)**	**No (n = 463)**	
*Age (years)of respondent*			
≤30	48	183	
31-40	78	190	0.085
41+	26	90	
*Education*			
No education	42	222	
Primary	27	124	0.001
Secondary +	83	117	
*Occupation*			
Farming	36	227	
Service	36	55	0.001
Business	67	131	
Driving	13	50	
*Monthly income (BDT)*			
Up to 2,500	50	180	
2,501 – 4,000	48	192	0.001
4,001 – 5,500	34	45	
5,501+	20	46	
*Access to media*			
No	28	182	0.001
Yes	124	281	
*Number of living children*			
<1	57	203	
2	54	100	0.003
3+	41	160	
*Marital duration (years)*			
<5	37	140	
5 - 10	56	127	0.080
11+	59	196	
*Wife work outside*			
Yes	131	43	0.779
No	39	420	
*Approval of family planning*			
Yes	149	436	0.055
No	3	27	
*Currently using family-planning methods*			
Yes	97	291	0.817
No	55	172	

χ^2^ test was performed to find the association. BDT: Bangladeshi Taka.

Table 4 shows the results of logistic regression analysis, which was performed for identifying the factors affecting the involvement of males in reproductive healthcare services. The fitness of model was significant; chi-square was 81.472 (p < 0.001), and -2Log likelihood was 604.049. Men having secondary and higher-level education were more likely to be involved in reproductive healthcare services than men who had no or primary education. Men who were paid employees were more likely to be involved in reproductive healthcare services compared to farming professionals. Men who had two children had higher odds of involvement in reproductive healthcare services than those with no or one children whereas men having three or more children had lower odds of involved in reproductive healthcare services. Men with the marital duration of 5–10 years were significantly more likely to be involved in reproductive healthcare services than those with the marital duration of less than five years. Men who had access to media were more likely to be involved in reproductive healthcare services than their counterparts.

**Table 4:** Logistic regression analysis on involvement of male in reproductive healthcare services

**Independent variable**	**β**	**p value**	**OR**	**95% CI for Exp (β)**	
				**Lower**	**Upper**
Age (years)*	−0.007	0.764	0.993	0.951	1.058
*Educational level*					
No education	Reference				
Up to primary	-0.039	0.895	0.962	0.541	1.711
Secondary+	0.925	0.001	2.523	1.439	4.424
*Occupation*					
Farming	Reference				
Service	0.912	0.005	2.489	1.3 14	4.714
Business	0.814	0.003	2.256	1.3 14	3.875
Driving	0.245	0.521	1.278	.065	2.699
Monthly income (BDT)*	0.000	0.117	1.000	1.000	1.000
Access to media					
No	Reference				
Yes	0.647	0.015	1.910	1.136	3.213
*Number of living children*					
≤1	Reference				
2	0.659	0.043	1.934	1.021	3.664
3+	0.075	0.851	1.078	0.492	2.363
*Duration (years)of marital life*					
<5	Reference				
5-10	0.548	0.081	1.730	0.934	3.202
11+	0.436	0.292	7.547	0.687	3.481
*Wife works outside*					
No	Reference				
Yes	0.164	0.653	1.179	0.575	2.415
*Approval of family planning*					
No	Reference				
Yes	0.876	0.181	2.402	0.664	8.688
*Currently using family- planning methods*					
No	Reference				
Yes	−0.019	0.934	0.981	0.622	1.548
−2log likelihood	604.049	0.000			
Model chi-square	81.472	0.000	0.039		
Constant	−3.236				

*In this model, age and monthly income (BDT) were considered as continues variables.

## Discussion

In reproductive health matters, most people viewed women as the target group, and little attention is given to the role of men. However, in patriarchal society where decisions are largely made by men, the needs to include them in all matters that require joint spousal decisions are crucial to achieving the reproductive health goals. This paper aimed to determine the factors that influence the involvement of males in reproductive healthcare. The study results mainly revealed that when men had a higher level of education, their involvement in reproductive healthcare was more. The result is comparable to the study conducted in Nigeria where men without formal education are likely to more conservative outlook towards family life [13]. Men are more exposed to radio, TV, newspapers, and diversified personal communication than women as men generally have more free time, more education, more disposable income, and, in many cultures, more freedom of movement than women [14]. Men who have exposure on mass media have effects in changing their attitudes to use of family planning and their spousal communications improve. This exposure of men obviously increased contraceptive-use and following the use of mass media has other behavioral change [15]. The results of the present study showed that the majority 66% of the men discussed the reproductive health-related matters with their wives and accompanied their wives for seeking reproductive healthcare services. The proportion of couples in this study are currently using any contraceptive methods was 63%. The results also revealed that most married couples were approving family-planning methods (95%) in achieving the reproductive health benefits, which indicates that men knowledge on family planning was high. A study was conducted in Ethiopia showed that 96% of married men approved at least one method of family planning [16], which is similar with our study.

The results of logistic regression analysis again revealed that men having more education, higher income level, and those who access to media were more likely to be involved in reproductive healthcare services. The logistic regression results further documented that men with the marital duration of 5–10 years were more likely to be involved in reproductive healthcare services compared to their counterparts. The longer marital duration increases men’s participation in reproductive healthcare services.

It is, thus, a very crucial area that needs continuous strengthening and increasing male’s participation in reproductive health services to reduce the maternal pregnancy related risks. This male participation in reproductive health issues also leads to better understanding between husbands and wives, which would reduce unwanted pregnancies and the unmet needs for family planning. Increasing the perceptions of men through involvement in various reproductive activities is believed to produce synergistic effects that could be a greater integration of reproductive health matters with the MDGs. The growing private sector in Bangladesh will have the greater scope to provide men-friendly reproductive health services, which deserves due attention more. Finally, it could be suggested that more pragmatic and target-oriented program are be required to increase the involvement of men in reproductive health matters in Bangladesh.

### Ethical consideration

This study was approved by the National Ethics Committee of the Bangladesh Medical Research Council. Written informed consent was obtained from the all participants. Ethics has been respected throughout the whole study period.

## Competing interests

The authors declare that they have no competing interests.

## Authors’ contributions

**MS** contributed his intellectual ability to conception and design of the research analysis and interpretation of data; drafting the article and revising it critically for important intellectual content; and final approval of the version to be published. **SJM, AA and HAC** participated on drafting the article and revising it critically for important intellectual content; and final approval of the version to be published. **RK** revises the manuscript for important intellectual content. **KA** works on analysis and interpretation of data; and final approval of the version to be published. All authors’ read and approved the final manuscript.

## References

[B1] Odu OO Jadunola KTI Parakoyi DB Reproductive behaviour and determinants of fertility among men in a semi-urban Nigerian community Community Primary Health Care 2005 17 1 13 19

[B2] Reproductive Health Outlook, PATH (RHO) Men and reproductive health 2003 http://www.igwg.org/igwg_media/rhowebsite2004.pdf

[B3] Kaushalendra KS Shelah SB Amy OT Husbands’ reproductive health, knowledge, attitudes and behavior in Uttar Pradesh, India Stud Fam Plann 1998 29 4 388 399 10.2307/172251 9919632

[B4] Estborn B Gendering men shared concern. Women’s empowerment base. Planning and sexual health. Technical Report, No. 28 1995

[B5] United Nations Population Fund Male involvement in reproductive health, including family http://snap3.uas.mx/RECURSO1/unfpa/data/docs/unpf0074.pdf

[B6] Khan ME Khan MI Mukerjee N Men’s attitude towards sexuality and their sexual behaviour: observations from rural Gujarat Proceedings of the National Seminar on Male Involvement in Reproductive Health and Contraception: IUSSP, April 30 - May 2, Baroda 1997

[B7] Rosliza AM Majdah M Male participation and sharing of responsibility in strengthening family planning activities in malaysia Malaysian Journal of Public Health Medicine 2010 10 1 23 27

[B8] Berhane Y Male involvement in reproductive health Ethiopian J Health Dev 2006 20 3 135 136

[B9] Ezeh AC The influence of spouses on each other's contraceptive attitudes in Ghana Stud Fam Plann 1993 24 3 163 174 10.2307/2939231 8351697

[B10] Fakeye O Babaniyi O Reasons for non-use of family planning methods at Ilorin, Nigeria; male opposition and fear of methods Trop Doct 1989 1 114 117 2773048 10.1177/004947558901900307

[B11] Central Statistics Agency [Ethiopia] and ORC Macro Ethiopia Demographic and Health Survey 2005 Addis Ababa, Ethiopia and Calverton, Maryland, USA: Central Statistical Agency and ORC Macro

[B12] Global Health Merson MH, Black RE Diseases, Programs, System and Policies 2012 Third Canada: Jones & Bartlett Learning

[B13] Isiugo-Abanihe UC Reproductive motivation and family size preferences among Nigerian men Stud Fam Plann 1992 23 3 211 215 7940620

[B14] Dudgeon MR Inhorn MC Men’s influences on women’s reproductive health: medical anthropological perspective Soc Sci Med 2004 59 1379 1395 10.1016/j.socscimed.2003.11.035 15246168

[B15] Haile A Enqueselassie F Influence of women's autonomy on couple's contraception use in Jimma town, Ethiopia Ethiop J Health Dev 2006 20 3 145 151

[B16] Mullick S Kunene B Wanjiru M Involving men in maternity care: health service delivery issues Agenda Special Focus, pp 2005 124 135

